# Risk of sepsis in patients with primary aldosteronism

**DOI:** 10.1186/s13054-018-2239-y

**Published:** 2018-11-21

**Authors:** Chieh-Kai Chan, Ya-Hui Hu, Likwang Chen, Chin-Chen Chang, Yu-Feng Lin, Tai-Shuan Lai, Kuo-How Huang, Yen-Hung Lin, Vin-Cent Wu, Kwan-Dun Wu, TAIPAI Study Group, Vin-Cent Wu, Chieh-Kai Chan, Jui-Hsiang Lin, Wei-Jie Wang, Che-Hsiung Wu, Ya-Hui Hu, Leay Kiaw Er, Chia-Hui Chang, Ya-Li Chang, Yao-Chou Tsai, Chih-Chin Yu, Yen-Hung Lin, Yi-Luwn Ho, Hung-Wei Chang, Lian-Yu Lin, Fu-Chang Hu, Chin-Chen Chang, Kao-Lang Liu, Shuo-Meng Wang, Kuo-How Huang, Shih-Chieh Jeff Chueh, Shih-Cheng Liao, Ching-Chu Lu, Ruoh-Fang Yen, Kwan-Dun Wu

**Affiliations:** 1 Department of Internal Medicine, National Taiwan University Hospital, Hsin-Chu branch, Hsin Chu, Taiwan; 2 Department of Internal Medicine, National Taiwan University Hospital, Taipei, Taiwan; 3 Division of Endocrinology and Metabolism, Department of Internal Medicine, Taipei Tzu Chi Hospital, The Buddhist Medical Foundation, Taipei, Taiwan; 4 Institute of Population Health Sciences, National Health Research Institutes, Miaoli, Taiwan; 5 Department of Medical Imaging, National Taiwan University Hospital, Taipei, Taiwan; 6 Department of Urology, National Taiwan University Hospital, Taipei, Taiwan; 7 TAIPAI, Taiwan Primary Aldosteronism Investigation (TAIPAI) Study Group, Taipei, Taiwan; 8 Graduate Institute of Clinical Medicine, College of Medicine, National Taiwan University, Taipei, Taiwan

**Keywords:** Primary aldosteronism, Hypertension, Sepsis, Oxidative stress, Chronic inflammation, Glucocorticoid, Taiwan Primary Aldosteronism Investigation

## Abstract

**Background:**

The interaction between hyperaldosteronism and immune dysfunction has been reported and glucocorticoid co-secretion is frequently found in primary aldosteronism (PA). The aforementioned conditions raise the possibility of the infection risk; however, clinical episodes of sepsis have not been reported in PA.

**Methods:**

Using Taiwan’s National Health Insurance Research Database between 1997 and 2009, we identified PA and aldosterone-producing adenoma (APA) matched with essential hypertension (EH) at a 1:1 ratio by propensity scores. The incidences of sepsis and mortality after the index date were evaluated, and the risk factors of outcomes were identified using adjusted Cox proportional hazards models and taking mortality as a competing risk.

**Results:**

We enrolled 2448 patients with PA (male, 46.08%; mean age, 48.4 years). There were 875 patients who could be ascertained as APA. Taking mortality as the competing risk, APA patients had a lower incidence of sepsis than their matched EH patients (hazard ratio (HR) 0.29; *P* < 0.001) after target treatments. Patients receiving adrenalectomy showed a benefit of decreasing the risk of sepsis (PA vs EH, HR 0.14, *P* = 0.001; APA vs EH, HR 0.16, *P* = 0.003), but mineralocorticoid receptor antagonist treatment may differ. Compared with matched control cohorts, patients with APA had a lower risk of all-cause mortality (PA, adjusted HR 0.84, *P* = 0.050; APA, adjusted HR 0.31, *P* < 0.001) after target treatments.

**Conclusions:**

Our study demonstrated that patients with PA/APA who underwent adrenalectomy could attenuate the risk of sepsis compared with their matched EH patients. We further found that APA patients with target treatments could decrease all-cause mortality compared with EH patients.

**Electronic supplementary material:**

The online version of this article (10.1186/s13054-018-2239-y) contains supplementary material, which is available to authorized users.

## Background

Primary aldosteronism (PA), characterized by an inappropriate production of aldosterone, is the most common form of secondary hypertension [1, 2]. Current studies have demonstrated that aldosterone oversecretion is not only related to fluid overload and hypokalemia but also resulted in cardiovascular and renal damage [3, 4].

Hyperaldosteronism is associated with proinflammatory immune dysregulation, such as the release of proinflammatory cytokines [5] and generating oxidative stress [6]. Systemic aldosterone infusion leads to oxidative stress and inflammation in the rat myocardium [7]. In human leucocytes, mineralocorticoid receptor (MR) expression has been reported in the CD34^+^ hematopoietic progenitor, and in peripheral blood T and B lymphocytes, monocytes, and neutrophils turning them sensitive to aldosterone stimulation [8]. On the other hand, recent studies [9, 10] have reported that glucocorticoid oversecretion was found in PA. In Cushing’s syndrome, glucocorticoid oversecretion can affect both the cellular and humoral components of the innate immune system [11]. The aforementioned condition raised the possibility of immune dysregulation in PA and it is reasonable to hypothesize that PA was associated with a higher risk of severe infection than other disease with immune dysregulation status [11]. However, there were few reports about the outcome of sepsis in PA patients. In addition, it is still unclear whether adrenalectomy for aldosteronism also leads to relevant hypocortisolism for a variable stress condition, especially during sepsis.

To study the effect of aldosterone on immune dysfunction, it is important to evaluate the risk of sepsis and septic shock among PA patients and the benefit of targeting treatments to PA patients. Therefore, we took advantage of the National Health Insurance registration database to conduct a large longitudinal population study about the correlation between sepsis and PA.

## Methods

### Data source

Our study used a longitudinal database created by the National Health Research Institutes (NHRI) through extracting original Taiwan National Health Insurance (TNHI) data (23.12 million insured population in 2009) for all patients who had ever had a PA diagnosis from 1997 to 2009. This database enabled us to investigate each patient from the day of PA diagnosis until the day of death, NHI disenrollment, or the end of 2010, whatever occurred first. Disenrollment from Taiwan’s NHI is rarely observed because the NHI is universal and compulsory for Taiwan’s citizens and foreigners with residence permits. For a person who should enroll in the NHI, disenrollment only occurs when the person is going to be overseas for at least 6 months or has been missing for at least 6 months. The NHI covers a broad spectrum of health services. As indicated by the Taiwan National Health Interview Survey data, the NHI offers healthcare in almost all outpatient visits and hospital stays for Taiwan’s citizens. Thus, our database includes nearly all medical records for each patient.

**Fig. 1:**
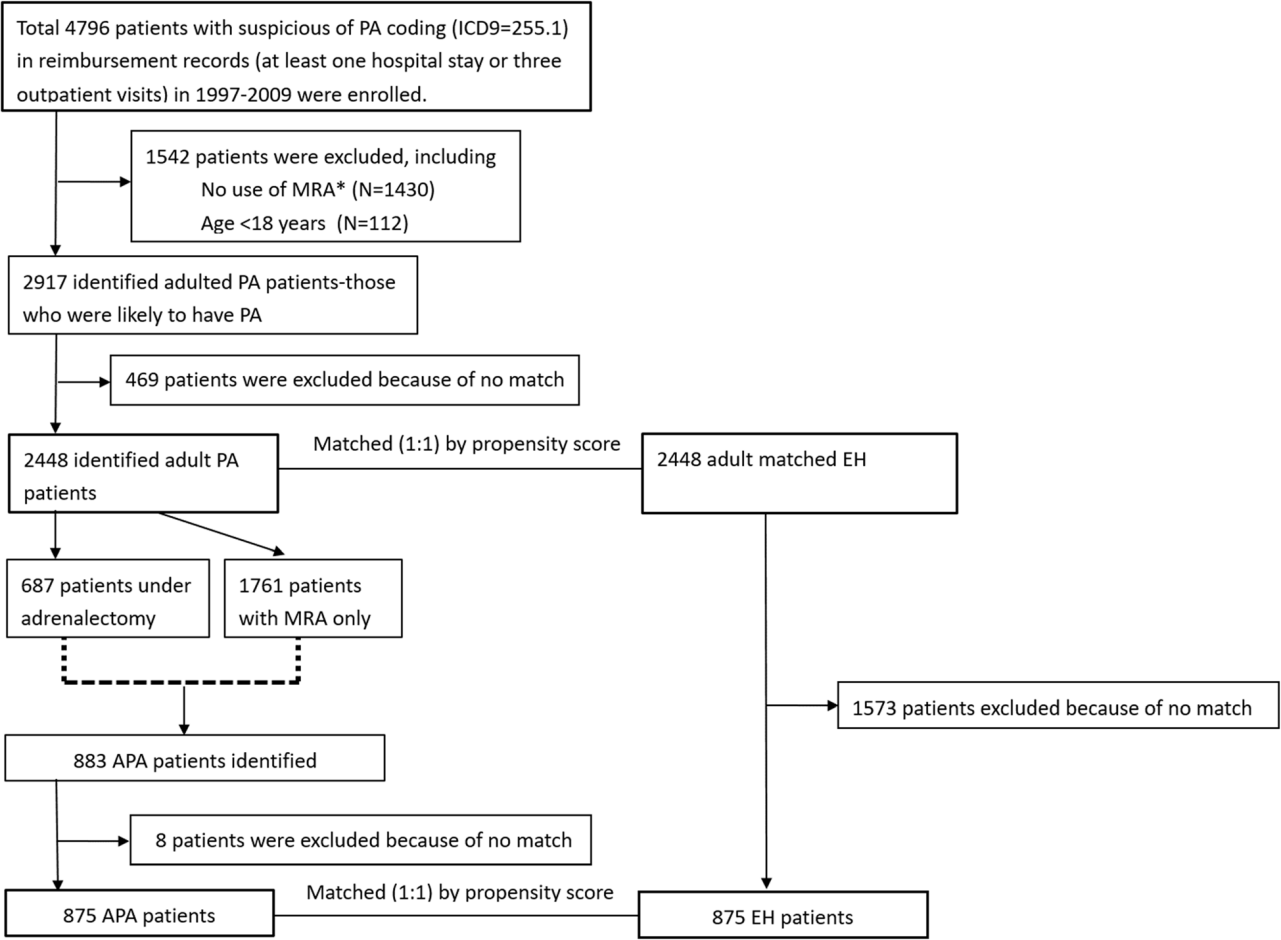
Flowchart of participants. *Patients who did not use MRA during the year before or 2 years after the first PA coding. *APA* aldosterone-producing adenoma, *EH* essential hypertension, *ICD* International Classification of Diseases, *MRA* mineralocorticoid receptor antagonist, *PA* primary aldosteronism

We used a validated algorithm to identify PA patients, and enrolled patients aged ≥18 years at the time of first medical record of PA (9th edition of International Classification of Diseases, ICD-9 code 255.1). The administrative data on diagnosis of and identifying PA patients has been well validated [12]. Figure 1 depicts the algorithm for selecting study patients. We only enrolled patients who used mineralocorticoid receptor antagonist (MRA) (belonging to the Anatomical Therapeutic Chemical (ATC) classification system, class C03D), because this additional condition assured high values for both sensitivity and positive predictive value [12, 13]. Patients with aldosterone-producing adenoma (APA) were enrolled from patients with PA who had undergone adrenalectomy or received a diagnosis of adrenal adenoma [12–14]. Each PA and APA patient was then matched with one patient with essential hypertension (EH) (without PA diagnosis and had used antihypertensive drugs) according to age, sex, and propensity score. We further separated PA and APA patients into two groups according to the target treatments, as those who received adrenalectomy or only MRA.

### Baseline characteristics

Concomitant medication data associated with blood pressure and outcome were recorded. The inotropic agents used during sepsis were also identified. The NHRI claims data regarding adrenalectomy and medications are reliable because they were copied on the basis of the NHI procedure and drug codes that were tied to the NHRI reimbursement system with auditing. The indication and guideline for hypertensive management in Taiwan have been proposed and revised by the Taiwan Hypertension Society [15]. Briefly, a diagnostic algorithm was proposed, emphasizing the ESH/ESH joint hypertension guidelines suggestion to loosen BP targets to < 140/90 mmHg for all patients [15].

### Outcomes

The main study outcome was incident sepsis after the index date. The identification of patients with sepsis was similar to that used by Lai et al. [16], who selected all acute-care hospitalizations with ICD-9-CM codes for both bacterial or fungal infection processes and a diagnosis of acute organ dysfunction. We used codes for acute organ dysfunction, as modified by Shen et al. [17] (Additional file 1). Sepsis was defined according to the American College of Chest Physicians/Society of Critical Care Medicine (ACCP/SCCM) as systemic inflammatory syndrome in response to infection, associated with acute organ dysfunction [18]. Septic shock was defined as sepsis with inotropic agents used during index episodes (Additional file 2). For patients with more than one hospital admission for sepsis during the study period, we only include the first episode of sepsis. We also recorded the risk of the all-cause mortality as the secondary outcome indicators. If a patient had not encountered any event at the time of NHI disenrollment or the end of 2010, we categorized this patient as a censored observation in our survival analysis.

To develop and test a standardized method for assessing the quality (completeness and accuracy) of clinical databases, a directory of clinical databases (DocDat) has been established [19]. In our database, most criteria had achieved Level 3 and Level 4, which indicated that our database had good quality and strength.

### Statistical analysis

Continuous variables were expressed as a mean ± standard deviation and categorical variables were expressed as a percentage. We matched PA /APA patients to their EH patients using a greedy matching algorithm with a caliper width of 0.2 standard differences (SDs) of the log odds of the estimated propensity score. The sampling ratios between patients of PA to EH and of APA to EH were 1:1 [20, 21]. Statistical significance was defined as two-sided *P* < 0.05.

In various subsequent multivariable models for analyzing outcomes, we also took into account the propensity score for the PA diagnosis in order to minimize residual confounding effects in the matching process (Additional file 3). Additional adjustment in these models included control for direct effects from age, gender, concomitant medications (except inotropic agents), and comorbidities as presented in Table 1. Cox regression models with a conditional approach and stratification were used to calculate hazard ratios (HRs) and 95% confidence intervals (CIs) for the risk of sepsis, mortality, and septic shock in each group. In further parametric modeling with regard to factors associated with outcome, we adopted three modeling methods: simple Cox regression, multivariable Cox regression, and competing risk regression. Because of the high mortality rate and sepsis rate in patients of old age and male patients, competing risk regression using the Fine and Gray model by considering the subdistribution hazard was also performed [22].

**Table 1:** Comparison of characteristics between PA and EH patients, for the whole PA cohort and for the APA subgroup only

Category	Matched EH/PA			Matched EH/APA		
	EH (*n* = 2448)	PA (*n* = 2448)	*p*	EH (*n* = 875)	APA (*n* = 875)	*p*
Propensity score	−3.92 ± 1.72	− 3.92 ± 1.72	0.995	−0.95 ± 0.38	−0.95 ± 0.38	0.993
Male gender	1146 (46.81%)	1128 (46.08%)	0.626	375 (42.86%)	377 (43.09%)	0.961
Age	48.40 ± 13.52	48.40 ± 13.52	0.999	46.62 ± 12.77	46.31 ± 10.55	0.829
Comorbidity						
Congestive heart failure	11 (0.45%)	8 (0.33%)	0.647	2 (0.23%)	3 (0.34%)	0.999
Cerebrovascular disease	105 (4.29%)	101 (4.13%)	0.831	29 (3.31%)	41 (4.69%)	0.179
CKD	29 (1.18%)	43 (1.76%)	0.122	8 (0.91%)	7 (0.80%)	0.999
COPD	91 (3.72%)	92 (3.76%)	0.999	19 (2.17%)	17 (1.94%)	0.867
Coronary artery disease	1 (0.04%)	5 (0.20%)	0.218	N/A	N/A	N/A
Dementia	10 (0.41%)	11 (0.45%)	0.999	1 (0.11%)	2 (0.23%)	0.999
Diabetes mellitus	208 (8.50%)	203 (8.29%)	0.837	55 (6.29%)	66 (7.54%)	0.346
Hemiplegia	7 (0.29%)	7 (0.29%)	0.999	1 (0.11%)	4 (0.46%)	0.374
Liver disease	107 (4.37%)	100 (4.08%)	0.670	25 (2.86%)	32 (3.66%)	0.419
Peptic ulcer	154 (6.29%)	138 (5.64%)	0.365	37 (4.23%)	48 (5.49%)	0.266
Peripheral vascular disease	8 (0.33%)	7 (0.29%)	0.999	3 (0.34%)	2 (0.23%)	0.999
Rheumatoid arthritis	15 (0.61%)	6 (0.25%)	0.078	2 (0.23%)	2 (0.23%)	0.999
Solid tumor	43 (1.76%)	49 (2.00%)	0.599	14 (1.60%)	16 (1.83%)	0.854
SLE	2 (0.08%)	2 (0.08%)	0.999	1 (0.11%)	1 (0.11%)	0.999
AF	10 (0.41%)	10 (0.41%)	0.999	4 (0.46%)	2 (0.23%)	0.687
Dyslipidemia	203 (8.29%)	194 (7.92%)	0.675	76 (8.69%)	65 (7.43%)	0.380
Alzheimer disease	0 (0.00%)	1 (0.04%)	0.999	0 (0.00%)	1 (0.11%)	0.999
Parkinson disease	14 (0.57%)	10 (0.41%)	0.540	4 (0.46%)	1 (0.11%)	0.374
Hypertensive drugs by categories						
Alpha-blocker	171 (6.99%)	187 (7.64%)	0.410	75 (8.57%)	78 (8.91%)	0.866
ACEI or ARB	1014 (41.42%)	997 (40.73%)	0.642	417 (47.66%)	403 (46.06%)	0.533
Beta-blocker	1165 (47.59%)	1157 (47.26%)	0.841	465 (53.14%)	473 (54.06%)	0.737
Calcium-channel blocker	1556 (63.56%)	1528 (62.42%)	0.424	631 (72.11%)	626 (71.54%)	0.832
Diuretic	1091 (44.57%)	1106 (45.18%)	0.687	416 (47.54%)	385 (44.00%)	0.150
Other concomitant medications						
Aspirin	62 (2.53%)	58 (2.37%)	0.782	20 (2.29%)	21 (2.40%)	0.999
Clopidogrel	7 (0.29%)	8 (0.33%)	0.999	2 (0.23%)	5 (0.57%)	0.452
Ticlopidine	10 (0.41%)	10 (0.41%)	0.999	2 (0.23%)	3 (0.34%)	0.999
Warfarin	16 (0.65%)	10 (0.41%)	0.326	9 (1.03%)	3 (0.34%)	0.145
PPI	79 (3.23%)	65 (2.66%)	0.271	24 (2.74%)	20 (2.29%)	0.647
H_2_ blocker	190 (7.76%)	176 (7.19%)	0.480	63 (7.20%)	61 (6.97%)	0.926
Statin	119 (4.86%)	112 (4.58%)	0.686	38 (4.34%)	36 (4.11%)	0.906
NSAID	1136 (46.41%)	1114 (45.51%)	0.547	408 (46.63%)	393 (44.91%)	0.502
Steroid	182 (7.43%)	186 (7.60%)	0.871	67 (7.66%)	58 (6.63%)	0.458
SSRI	58 (2.37%)	42 (1.72%)	0.129	19 (2.17%)	15 (1.71%)	0.604
Nitrate	4 (0.16%)	5 (0.20%)	0.999	1 (0.11%)	0 (0.00%)	0.999
Inotropic agents used during index hospitalization						
Dopamine	3 (0.12%)	3 (0.12%)	0.999	2 (0.23%)	1 (0.11%)	0.999
Norepinephrine	17 (0.69%)	16 (0.65%)	0.999	7 (0.80%)	2 (0.23%)	0.179
Vasopressin	9 (0.37%)	0 (0.00%)	0.004	4 (0.46%)	0 (0.00%)	0.125
Epinephrine	39 (1.59%)	39 (1.59%)	0.999	14 (1.60%)	2 (0.23%)	0.004
Outcome						
Sepsis	87 (3.55%)	104 (4.25%)	0.238	29 (3.31%)	9 (1.03%)	0.001
Septic shock	58 (2.37%)	57 (2.33%)	0.999	19 (2.17%)	6 (0.69%)	0.014
Mortality	273 (11.15%)	236 (9.64%)	0.092	84 (9.60%)	28 (3.20%)	< 0.001

All data presented as number (%), except mean age, Charlson Comorbidity Index Score, and propensity score

*ACEI* angiotensin-converting enzyme inhibitor, *AF* atrial fibrillation, *APA* aldosterone-producing adenoma, *ARB* angiotensin II receptor blocker, *CKD* chronic kidney disease, *COPD* chronic obstructive pulmonary disease, *EH* essential hypertension, *NA* nonavailable, *NSAID* nonsteroidal antiinflammatory drug, *PA* primary aldosteronism, *PPI* proton-pump inhibitor, *SLE* systemic lupus erythematosus, *SSRI* selective serotonin reuptake inhibitor

We used R software, version 2.8.1 (Free Software Foundation, Inc., Boston, MA, USA); competing risk analysis was performed with Stata/MP version 12 (Stata Corporation, College Station, TX, USA). Two-sided *P* < 0.05 was considered statistically significant.

## Results

### Characteristics of the study population

After propensity score matching, 2448 patients with PA were matched to the 2448 patients with EH (Fig. 1). Among these 4896 patients, only 22 patients (10 in PA group and 12 in EH group) disenrolled from the NHI before the end of 2010 and remained alive at the time of NHI disenrollment. These 22 patients were treated as censored observations with regard to outcome. The propensity score, gender, age, comorbidities, and ascertained drugs were not significantly different between the two cohorts. The mean age of the PA patients was 48.4 years at the time of PA diagnosis, and the proportion of men was 46.1% (Table 1). There were 875 patients who could be ascertained as APA and were matched with 875 EH patients (Fig. 1).

### Long-term risks of sepsis and mortality between PA and matched EH patients

The PA cohort had a similar sepsis rate as the EH cohort (4.25% vs 3.55%, *P* = 0.238) during a mean follow-up period of 4.28 years. The mortality rate in the PA and EH cohorts was similar (9.64% vs 11.15%, *P* = 0.092) (Table 1).

The adjusted HR for developing sepsis among the PA cohort after target treatments, relative to the EH cohort, was 1.18 (*P* = 0.250), taking into account the competing effects of death, corresponding to an absolute detrimental risk of 1.0 per 1000 person-years. The competing risk of mortality-adjusted HR for developing septic shock between the PA and EH cohorts was 0.96 (*P* = 0.850) (Table 2). The adjusted HR after target treatments for developing morality among the PA cohort, relative to the EH cohort, was 0.84 (*P* = 0.050) (Table 1).

**Table 2:** Incidence and risks for outcomes of interest between PA patients and their EH matches, for the whole PA cohort and for the APA subgroup only

Outcome							Crude		Adjusted^a^		Competing risk^b^	
	Events	Person-years	Incidence rate per 1000 person-years	Events	Person-years	Incidence rate per 1000 person-years	Hazard ratio (95% CI)	*p*	Hazard ratio (95% CI)	*p*	Hazard ratio (95% CI)	*p*
	PA			EH			PA vs EH					
Sepsis	104	13,652.95	7.6	87	13,144.31	6.6	1.15 (0.87, 1.53)	0.332	1.17 (0.88, 1.55)	0.286	1.18 (0.89, 1.57)	0.250
Septic shock	57	13,772.55	4.1	58	13,211.21	4.4	0.94 (0.65, 1.36)	0.754	0.96 (0.66, 1.38)	0.816	0.96 (0.67, 1.39)	0.850
All-cause mortality	236	13,836.93	17.1	273	13,268.23	20.6	0.83 (0.70, 0.99)	0.035	0.84 (0.71, 1.00)	0.050	N/A	N/A
	APA			EH			APA vs EH					
Sepsis	9	5550.02	1.6	29	5104.02	5.7	0.28 (0.13, 0.59)	0.001	0.28 (0.13, 0.59)	0.001	0.29 (0.14, 0.61)	< 0.001
Septic shock	6	5556.85	1.1	19	5122.85	3.7	0.28 (0.11, 0.71)	0.007	0.28 (0.11, 0.71)	0.007	0.30 (0.12, 0.75)	0.010
All-cause mortality	28	5567.03	5	84	5141.56	16.3	0.31 (0.20, 0.47)	< 0.001	0.31 (0.20, 0.47)	< 0.001	N/A	N/A

*APA* aldosterone-producing adenoma, *CI* confidence interval, *EH* essential hypertension, *NA* nonavailable, *PA* primary aldosteronism

^*^Multivariate Cox regression model selected covariates from all variables presented in Table 1 by a stepwise procedure

^b^Competing risk regression model included age, gender, and propensity score as covariates

As shown in Table 3, patients with PA who received adrenalectomy had an attenuated risk of developing sepsis (adjusted HR 0.14, *P* = 0.001) compared to matched EH patients analyzed by the multivariate Cox regression model, even after accounting for the competing risk of death (competing hazard ratio (cHR) 0.14, *P* = 0.001; we used cHR as the hazard ratio after accounting for the competing risk of death). A similar result was also found in septic shock (cHR 0.14, *P* = 0.005) after accounting for the competing risk of death. Lower risk of mortality was also found in PA patients after adrenalectomy (adjusted HR 0.21, *P* < 0.001). However, PA patients with MRA had a higher risk of developing sepsis (cHR 1.49, *P* = 0.004) after taking death as a competing risk than patients with EH. Additional file 4 presents the risks from sepsis and death between PA patients and their EH matches without loss of follow-up patients, for the patients only by target treatments (*N* = 4874).

**Table 3:** Comparison of risks from sepsis and death between PA patients and their EH matches, for the whole PA cohort and for the APA subgroup only by target treatments

Outcome	Adrenalectomy						MRA					
	Crude		Adjust^a^		Competing risk^b^		Crude		Adjusted^a^		Competing risk^b^	
	Hazard ratio (95% CI)	*p*	Hazard ratio (95% CI)	*p*	Hazard ratio (95% CI)	*p*	Hazard ratio (95% CI)	*p*	Hazard ratio (95% CI)	*p*	Hazard ratio (95% CI)	*p*
	PA vs EH						PA vs EH					
Sepsis	0.12 (0.04,0.39)	< 0.001	0.13 (0.04,0.41)	0.001	0.14 (0.04,0.44)	0.001	1.53 (1.15,2.04)	0.004	1.52 (1.14,2.03)	0.004	1.49 (1.12,1.98)	0.006
Septic shock	0.12 (0.03,0.49)	0.003	0.13 (0.03,0.53)	0.005	0.14 (0.03,0.55)	0.005	1.25 (0.87,1.81)	0.231	1.24 (0.86,1.80)	0.249	1.22 (0.84,1.76)	0.300
All-cause mortality	0.20 (0.12,0.33)	< 0.001	0.21 (0.13,0.36)	< 0.001	N/A	N/A	1.06 (0.89,1.26)	0.526	1.05 (0.88,1.25)	0.606	N/A	N/A
	APA vs EH						APA vs EH					
Sepsis	0.14 (0.04,0.45)	0.001	0.14 (0.04,0.46)	0.001	0.16 (0.05,0.53)	0.003	0.55 (0.23,1.35)	0.193	0.55 (0.22,1.33)	0.184	0.44 (0.18,1.07)	0.071
Septic shock	0.14 (0.03,0.60)	0.008	0.14 (0.03,0.61)	0.009	0.16 (0.04,0.67)	0.012	0.59 (0.20,1.78)	0.352	0.57 (0.19,1.70)	0.314	0.46 (0.15,1.36)	0.160
All-cause mortality	0.25 (0.14,0.43)	< 0.001	0.25 (0.15,0.44)	< 0.001	N/A	N/A	0.42 (0.24,0.76)	0.004	0.40 (0.22,0.72)	0.002	N/A	N/A

*APA* aldosterone-producing adenoma, *CI* confidence interval, *EH* essential hypertension, *MRA* mineralocorticoid antagonist, *NA* nonavailable, *PA* primary aldosteronism

^a^Multivariate Cox regression model selected covariates from all variables presented in Table 1 by a stepwise procedure. This model constructed adrenalectomy, steroid, and potassium supplement for hypokalemia as time-varying covariates

^b^Competing risk regression model included age, gender, and propensity score as covariates

### Long-term risks of sepsis and mortality between APA and matched EH patients

A lower sepsis rate was found in the APA cohort than in their matched EH cohort (1.03% vs 3.31%, *P =* 0.001) after target treatments during a mean follow-up period of 5.56 years (Table 1).

The adjusted HR for developing sepsis among the APA cohort after target treatments, relative to the EH cohort, was 0.29 (*P* < 0.001) taking into account the competing effects of death (Table 2). Taking into account death as a competing risk, the adjusted HR for developing septic shock between the APA and EH cohorts was 0.30 (*P* = 0.010). The adjusted HR for developing mortality among the APA cohort, relative to the EH cohort, was 0.31 (*P <* 0.001) (Table 2).

As shown in Table 3, patients with APA with adrenalectomy had a lower risk of developing sepsis (cHR 0.16, *P* = 0.003) and mortality (adjusted HR 0.25, *P* < 0.001) than patients with EH. Inconsistent with the aforementioned result, a lower incidence was also found in developing septic shock (cHR 0.16, *P* = 0.012) by adjusting death as a competing risk in APA rather than EH patients. However, patients with APA with only MRA treatment did not benefit from a reduced incidence of sepsis (cHR 0.44, *P* = 0.071) and septic shock (HR 0.46, *P* = 0.160). Nonetheless, MRA treatment could attenuate the risk of mortality in APA patients (adjusted HR 0.40, *P* = 0.002).

**Fig. 2:**
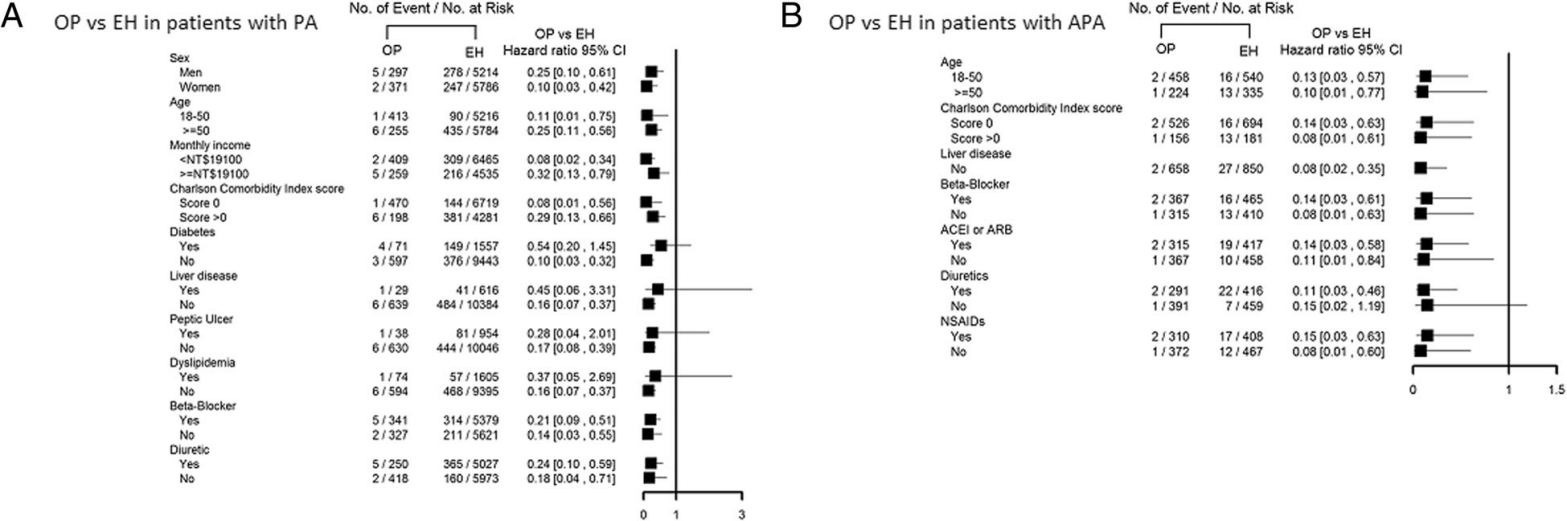
Risk of sepsis between PA (**a**) and APA (**b**) patients with adrenalectomy and their EH controls by participant characteristics. *ACEI* angiotensin-converting enzyme inhibitor, *APA* aldosterone-producing adenoma, *ARB* angiotensin receptor blocker, *CI* confidence interval, *EH* essential hypertension, *NSAID* nonsteroidal antiinflammatory drug, *OP* operation, *PA* primary aldosteronism

In the subgroup analysis by forest plot, the benefit of adrenalectomy on decreasing the risk of developing sepsis was also noted in patients with PA/APA compared with matched EH patients (Fig. 2).

**Fig. 3:**
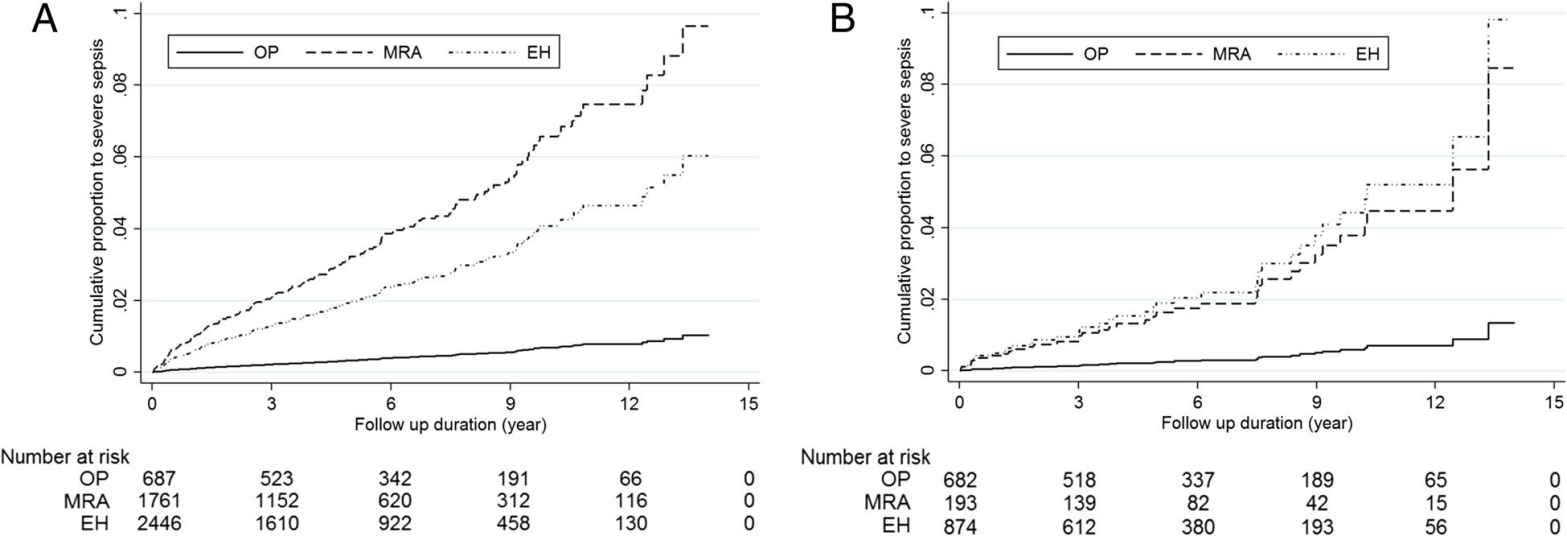
Proportional curve (adjusted with age, gender, and Charlson score) for sepsis in patients with adrenalectomy (OP), mineralocorticoid receptor antagonist (MRA) treatment, and essential hypertension (EH) during follow-up period by Cox regression model. Cumulative proportions to sepsis of PA (**a**) and APA (**b**) patients after target treatments (adrenalectomy or MRA treatment) and their EH controls by Cox proportional plot, taking mortality as a competing risk. Cox regression: (a) *P* < 0.001 (OP/MRA) and *P* = 0.001 (OP/EH); (b) *P* = 0.030 (OP/MRA) and *P* = 0.003 (OP/EH)

Furthermore, corresponding cumulative incidence curves that depict the accumulated proportions of incident sepsis showed a significantly lower rate in patients who received adrenalectomy than in patients under MRA treatment and EH patients after adjusting for age, gender, and Charlson morbidity index with death as a competing risk (Fig. 3a, b) (operation (OP) vs MRA, *P* < 0.001 and OP vs EH, *P* = 0.002 in PA group; OP vs MRA, *p* = 0.008 and OP vs EH, *P* = 0.001 in APA group).

## Discussion

In this population-based cohort study, we demonstrated the treatment benefit of adrenalectomy on attenuating the sepsis incidence among PA patients. However, the effect of MRA treatment on sepsis may differ. In addition, the risk of adrenalectomy-related adrenal insufficiency and subsequent shock during septic status is not significant. In line with the previous findings, our results further indicate that patients with PA/APA could benefit from MRA treatment or adrenalectomy in terms of long-term all-cause mortality.

### Immune system dysregulation with hyperaldosteronism

In addition to the regulatory role of the body fluid and electrolyte balance, prolonged exposure to hyperaldosterone causes cardiac and renal damage independent of high blood pressure levels [23, 24]. In light of recent concepts, chronic inflammatory disease could be attributed to sepsis [25, 26], and PA was regarded as a chronic inflammatory disease which may result from immune dysfunction.

Heart failure in patients with PA is attributed to the overproduction of oxidative stress and proinflammatory cytokine augmented by hyperaldosterone [6, 27]; this phenomenon was reported to be promoted by aldosterone and related to dysfunction of innate and adaptive immunity. On the other hand, proinflammatory cytokines could also play an important role. Krysiak and Okopien reported that higher levels of tumor necrosis factor alpha (TNF-α), interleukin 6 (IL-6), and interleukin 1 beta (IL-1β) from monocytes/macrophages and of interleukin 2 (IL-2), interferon gamma (IFN-γ), and TNF-α from lymphocytes were found in patients with APA than in patients with EH [28].

The possibility of glucocorticoid co-secretion in PA has been demonstrated by Arlt et al. [10], which could be an important reason for the higher sepsis risk in patients with PA. Glucocorticoid could alter and influence the cellular response to infection and the humoral component [29]. These conditions indicate that glucocorticoid excess is associated with immune dysfunction and is responsible for the susceptibility to infections.

Previous studies demonstrated that the role of the renin–angiotensin–aldosterone system according to increasing aldosterone level was noted after endotoxin infusion in an animal model [30]. In addition, Annane et al. [31] revealed that the patients with septic shock who received hydrocortisone plus fludrocortisone for 7 days had better long-term outcome than the placebo group. These studies showed the potential benefit of aldosterone and glucocorticoid in sepsis. However, long-term exposure to a high aldosterone level of PA patients may increase their sepsis risk resulting from chronic inflammation and immune dysfunction.

### Long-term outcome after target treatments to aldosteronism

In our study, adrenalectomy gains the benefit of ameliorating sepsis; however, MRA treatment may differ. PA patients exhibited decreased levels of transforming growth factor beta (TGF-β) and TNF-α, compared to normotensive patients; however, MRA treatment could only restore levels of TGF-β [32]. These results may, at least partly, explain the reason for the limited benefit of MRA treatment on decreasing the sepsis risk in patients with PA.

In addition, adrenalectomy might yield a therapeutic effect more rapidly [33], and it takes more than 1 year to ameliorate overhydration [34] and 6 years to observe reductions in left ventricular wall thickness and masses in PA patients after MRA treatment [35].

The benefit of adrenalectomy could also be explained by glucocorticoid co-secretion in PA. In recent studies, adrenalectomy in patients with APA could decrease glucocorticoid secretion, restore osteoporosis, attenuate adverse metabolic risk, and improve the quality of life [10, 22, 36, 37], which are attributed to decreased glucocorticoid levels in addition to mineralocorticoid excess.

### Adrenal dysfunction and sepsis/septic shock

Adrenal insufficiency was noted after unilateral adrenalectomy in patients with Cushing syndrome [38]; however, there were few reports about adrenal insufficiency in PA patients after unilateral adrenalectomy. Honda et al. [39] demonstrated that the cortisol level is sustained with an elevated adrenocorticotropic hormone (ACTH) level in APA patients who had status post unilateral adrenalectomy, which has raised the possibility of clinical adrenal insufficiency among them. Activation of the renin–angiotensin–aldosterone system (RAAS) is an important mechanism to preserve volume status and vascular tone [40]. However, high levels of inflammatory cytokines in patients with sepsis will inhibit adrenal cortisol synthesis [41]. Our results identified that APA patients who received unilateral adrenalectomy would not augment the risk of septic shock, as a result of adrenal insufficiency during severe stress.

### Adrenalectomy and MRA treatment gains the benefit of long-term all-cause mortality

The long-term different outcome of PA patients treated with adrenalectomy or MRA is still under debate. In our study, APA patients with adrenalectomy and MRA both had lower mortality than EH patients. We have found that adrenalectomy may reduce long-term all-cause mortality among PA patients, while MRA prescription may also help in this regard when its dosage is appropriate [13]. Interestingly, some small-scale studies have shown no difference between surgically and medically treated patients with PA in terms of the incidence of cardiovascular events [33, 42]. Our result raises an important, at least partial, account for the long-term improvement of mortality by sepsis in PA patients out of proportion to cardiovascular event improvement that only benefits substantially from adrenalectomy in the long-term follow-up.

### Study limitations

There were several limitations to our study. First, the diagnosis of PA, APA, and EH and the morbidities were identified by ICD-9-CM codes. Even though this study has a large patient size, some degree of misclassification might have affected the identification of patients. Second, as an observational study, there was still the possibility of selection bias and unmeasured confounding factors in our study. The results of our study could only demonstrate the association between aldosterone and sepsis, and it is hard to prove a causal relationship between aldosterone and sepsis and mortality in this study. We still need further investigation to clarify the relation between hyperaldosteronism and immune dysfunction. Third, the NHIRD did not contain information on several factors, including body mass index, nutritional status, and laboratory findings. However, a placebo-controlled randomized clinical trial regarding MRA or adrenalectomy over as many years as in our observational study is not possible nor is it ethically acceptable to conduct; our prospective follow-up study provided a valid alternative [43]. Fourth, this study used population-based health insurance registration data, where the diagnostic results were not ‘controlled’ as ideally as some single or multicenter clinical studies could have easily achieved.

## Conclusions

Our study demonstrated that patients with aldosteronism under adrenalectomy could attenuate the incidence of sepsis compared to patients with EH; however, patients receiving long-term MRA treatment may differ. Unilateral adrenalectomy of APA will not augment a risk of adrenal insufficiency under the stress of sepsis. We further found that APA patients under adrenalectomy and MRA treatments both had a lower mortality rate than EH patients. Further investigation is therefore necessary to clarify the effect of aldosterone on immune dysfunction.

## Additional files

Additional file 1: Acute organ dysfunction codes for sepsis. (DOCX 18 kb)

Additional file 2: ATC codes of inotropic agents. (DOCX 15 kb)

Additional file 3: Risk factors for PA diagnosis to minimize residual confounding effects in matching process to EH. (DOCX 20 kb)

Additional file 4: Comparison of risks from sepsis and death between PA patients and their EH matches without loss of follow-up patients, for patients only by target treatments (*N* = 4874). (DOCX 18 kb)

## Abbreviations

ACTH: Adrenocorticotropic hormone
APA: Aldosterone-producing adenoma
cHR: Competing hazard ratio
CI: Confidence interval
EH: Essential hypertension
HR: Hazard ratio
IFN-γ: Interferon gamma
IL-1β: Interleukin 1 beta
IL-2: Interleukin 2
IL-6: Interleukin 6
MR: Mineralocorticoid receptor
MRA: Mineralocorticoid receptor antagonist
NHRI: National Health Research Institutes
OP: Operation
PA: Primary aldosteronism
RAAS: Renin–angiotensin–aldosterone system
TGF-β: Transforming growth factor beta
TNF-α: Tumor necrosis factor alpha
TNHI: Taiwan National Health Insurance
