# Supplementary material for: Risk of sepsis in patients with primary aldosteronism
Source: Crit Care. 2018 Nov 21;22:313. doi: 10.1186/s13054-018-2239-y (PMC6249889; doi:10.1186/s13054-018-2239-y)
Supplement: Supplementary file 1 — Acute organ dysfunction codes for sepsis. (DOCX 18 kb) [file 13054_2018_2239_MOESM1_ESM.docx]

Additional file 1. Acute organ dysfunction code for sepsis

| Organ dysfunction | Codes | Code description |
| --- | --- | --- |
| Cardiovascular | 458.0 | Hypotension, postural |
|  | 458.8 | Hypotension, specified type, not elsewhere classified |
|  | 458.9 | Hypotension, arterial, constitutional |
|  | 785.5 | Shock |
|  | 785.51 | Shock, cardiogenic |
|  | 785.59 | Shock, circulatory or septic |
|  | 796.3 | Hypotension, transient |
| Respiratory | 518.81 | Acute respiratory failure |
|  | 518.82 | Acute respiratory distress syndrome (ARDS) |
|  | 518.85 | ARDS after shock or trauma |
|  | 786.09 | Respiratory insufficiency |
|  | 799.1 | Respiratory arrest |
|  | 96.7 (96.71,96.72) | Ventilator management |
|  | 96.04 | Endotracheal intubation (emergency procedure) |
|  | 93.9 | Continuous positive airway pressure |
| Renal | 580.x | Acute glomerulonephritis |
|  | 584.x | Acute renal failure |
|  | 586 | Renal shutdown, renal failure unspecified |
|  | 39.95 | Hemodialysis |
| Hepatic | 570 | Acute hepatic failure or necrosis |
|  | 572.2 | Hepatic encephalopathy |
|  | 573.3 | Hepatitis (septic & not elsewhere classified) |
|  | 573.4 | Hepatic infarction |
| Neurologic | 293 | Transient organic psychosis |
|  | 348.1 | Anoxic brain injury |
|  | 348.3 | Encephalopathy, acute |
|  | 780.01 | Coma |
|  | 780.09 | Altered consciousness, unspecified |
|  | 89.14 | Electroencephalography |
| Hematologic | 286.2 | Disseminated intravascular coagulation |
|  | 286.6 | Purpura fulminans |
|  | 286.9 | Coagulopathy |
|  | 287.3-5 | Thrombocytopenia, primary, secondary or unspecified |
|  | 790.92 | Abnormal coagulation profile |
| Metabolic | 276.2 | Acidosis, metabolic or lactic |
