# Supplementary material for: Risk of sepsis in patients with primary aldosteronism
Source: Crit Care. 2018 Nov 21;22:313. doi: 10.1186/s13054-018-2239-y (PMC6249889; doi:10.1186/s13054-018-2239-y)
Supplement: Supplementary file 2 — ATC codes of inotropic agents. (DOCX 15 kb) [file 13054_2018_2239_MOESM2_ESM.docx]

Additional file 2. ATC codes of inotropic agent

| Inotropic agent | ATC code |
| --- | --- |
| Dopamine | WHO ATC/DDD N04B |
| Norepinephrine | WHO ATC/DDD C01CA03 |
| Bicarbonate | WHO ATC/DDD A02AH, B05CB04, B05XA02 |
| Vasopressin | WHO ATC/DDD C03XA, H01BA, B01BA01 |
| Atropine | WHO ATC/DDD A03BA01, S01FA01, A03CB03, N04AC01, N04AC30, S01FA05, A03BB02, A03CB04 |
| Epinephrine | WHO ATC/DDD A01AD01, B02BC09, C01CA24, R01AA14, R03AA01, S01EA01, R03AK01, S01EA51, C01CA03 |
