# Supplementary material for: Risk of sepsis in patients with primary aldosteronism
Source: Crit Care. 2018 Nov 21;22:313. doi: 10.1186/s13054-018-2239-y (PMC6249889; doi:10.1186/s13054-018-2239-y)
Supplement: Supplementary file 3 — Risk factors for PA diagnosis to minimize residual confounding effects in matching process to EH. (DOCX 20 kb) [file 13054_2018_2239_MOESM3_ESM.docx]

Additional file 3. The risk factors for the PA diagnosis in order to minimize residual confounding effects in the matching process to EH.

| Items | OR | lower 95% CI | upper  95% CI | *p* |
| --- | --- | --- | --- | --- |
| Age | 0.954 | 0.952 | 0.957 | <0.001 |
| Man | 0.786 | 0.723 | 0.854 | <0.001 |
| Charlson score | 0.862 | 0.817 | 0.909 | <0.001 |
| *Baseline comorbidities* |  |  |  |  |
| Congestive heart failure | 0.084 | 0.042 | 0.170 | <0.001 |
| Dyslipidemia | 0.580 | 0.492 | 0.683 | <0.001 |
| Diabetes mellitus | 0.569 | 0.480 | 0.674 | <0.001 |
| SLE | 0.127 | 0.031 | 0.523 | <0.01 |
| COPD | 0.676 | 0.540 | 0.846 | <0.001 |
| CVA | 0.690 | 0.556 | 0.858 | <0.001 |
| Chronic Kidney disease | 0.567 | 0.411 | 0.782 | <0.001 |
| Myocardial infarction | 0.385 | 0.157 | 0.945 | 0.037 |
| Atrial fibrillation | 0.505 | 0.267 | 0.957 | 0.036 |
| Peripheral vascular disease | 0.480 | 0.231 | 0.995 | 0.048 |
| Medications |  |  |  |  |
| Diuretics | 4.369 | 3.981 | 4.795 | <0.001 |
| CCB | 6.838 | 6.152 | 7.602 | <0.001 |
| Beta-blocker | 2.195 | 2.011 | 2.396 | <0.001 |
| ACEI and ARB | 2.236 | 2.028 | 2.466 | <0.001 |
| Alpha-blocker | 2.949 | 2.520 | 3.450 | <0.001 |
| Aspirin | 0.413 | 0.316 | 0.539 | <0.001 |
| NSAID | 0.800 | 0.736 | 0.869 | <0.001 |
| Plavix | 0.304 | 0.149 | 0.619 | 0.001 |
| H2 blocker | 0.684 | 0.583 | 0.802 | <0.001 |
| Statin | 0.655 | 0.529 | 0.812 | <0.001 |
| Warfarin | 0.409 | 0.214 | 0.780 | <0.01 |
| Ticlopidine | 0.490 | 0.260 | 0.923 | 0.027 |
| Steroid | 0.842 | 0.719 | 0.986 | 0.033 |
| SSRI | 0.716 | 0.524 | 0.976 | 0.035 |

Hosmer-Lemeshow goodness of fit [GOF] test p<0.001, AUC= 0.827

This logistic regression model was adjusted with age, gender, congestive heart failure, CVA, CKD, COPD, coronary artery disease, dementia, diabetes Mellitus, hemiplegia, liver disease, peptic ulcer, peripheral vascular disease, rheumatoid arthritis, solid tumor, SLE, atrial fibrillation, dyslipidemia, Alzheimer disease, Parkinson disease, alpha-blocker, ACEI /ARB, beta-blocker, CCB, diuretic agent, aspirin, clopidogrel, ticlopidine, warfarin, proton pump inhibitor, H2 blocker, statin, NSAID, steroid, SSRI, nitrate, dopamine, norepinephrine, vasopressin, epinephrine,

**Abbreviations:** ACEI, Angiotensin Converting Enzyme Inhibitors; ARB, Angiotensin receptor blocker; CCB, calcium channel blocker; CI, confidence interval; CKD, chronic kidney disease, COPD, chronic obstructive pulmonary; CVA, cerebrovascular accident; NSAID, nonsteroidal anti-inflammatory drugs; SLE, systemic lupus erythematosus; SSRI, selective serotonin reuptake inhibitors
