# Supplementary material for: Risk of sepsis in patients with primary aldosteronism
Source: Crit Care. 2018 Nov 21;22:313. doi: 10.1186/s13054-018-2239-y (PMC6249889; doi:10.1186/s13054-018-2239-y)
Supplement: Supplementary file 4 — Comparison of risks from sepsis and death between PA patients and their EH matches without loss of follow-up patients, for patients only by target treatments (N = 4874). (DOCX 18 kb) [file 13054_2018_2239_MOESM4_ESM.docx]

Additional file 4 Comparison of risks from sepsis and death between PA patients and their EH matches without loss of follow up patients, for the patients only by target treatments. (N=4874)

|  |  | | Adrenalectomy | | | |  | | MRA | | | |
| --- | --- | --- | --- | --- | --- | --- | --- | --- | --- | --- | --- | --- |
| Outcome |  | | Adjust* | |  | |  | | Adjust* | |  | |
|  |  |  | Hazard Ratio  (95% CI) | | p | |  | | Hazard Ratio  (95% CI) | | p | |
| Sepsis |  | 0.13 [0.04,0.41] | | 0.001 | |  | | 1.52 [1.14,2.02] | | 0.004 | |  |
| All-cause Mortality |  | 0.21 [0.13,0.36] | | <0.001 | |  | | 1.05 [0.88,1.25] | | 0.621 | |  |

**Abbreviations:** APA, aldosterone-producing adenoma; CI, confidence interval; EH, essential hypertension; MRA, mineralocorticoid antagonist; PA, primary aldosteronism.

* The multivariate Cox regression model selected covariates from all variables in Table1 by a stepwise procedure. This model constructed adrenalectomy, steroid and potassium supplement for hypokalemia as time varying covariates.
